# The iBLAD study: patient-reported outcomes in bladder cancer during oncological treatment: a multicenter national randomized controlled trial

**DOI:** 10.1186/s41687-023-00640-5

**Published:** 2023-10-09

**Authors:** Gry Assam Taarnhøj, Christoffer Johansen, Andreas Carus, Rikke Hedegaard Dahlrot, Line Hammer Dohn, Niels Henrik Hjøllund, Mark Bech Knudsen, Anders Tolver, Henriette Lindberg, Helle Pappot

**Affiliations:** 1 Department of Oncology, Copenhagen University Hospital, Rigshospitalet, Blegdamsvej 9, 2100 Copenhagen Ø, Denmark; 2 CASTLE: Cancer Survivorship and Treatment, Late Effects National Research Center, Blegdamsvej 58, 2100 Copehnagen Ø, Denmark; 3 Department of Oncology, Aalborg University Hospital, Aalborg, Denmark; 4 Department of Clinical Medicine, Aalborg University and Clinical Cancer Research Center, Hobrovej 18-22, 9000 Aalborg, Denmark; 5 Department of Oncology, Odense University Hospital, Odense C, Denmark; 6 Institute of Clinical Research, University of Southern Denmark, J.B. Winsløws Vej 4, 5000 Odense, Denmark; 7 Department of Oncology, Copenhagen University Hospital, Herlev Hospital, Borgmester Ib Juuls Vej 1, 2730 Herlev, Denmark; 8 AmbuFlex - Center for Patient-Reported Outcomes, Central Denmark Region, Gødstrup Hospital, Hospitalsparken 15, 7400 Herning, Denmark; 9 Department of Clinical Epidemiology, Aarhus University Hospital, Olof Palmes Allé 43-45, 8200 Aarhus N, Denmark; 10 Data Science Lab, Department of Mathematical Sciences, University of Copenhagen, Universitetsparken 5, 2100 Copenhagen Ø, Denmark

**Keywords:** Patient-reported outcomes, Bladder cancer, Urothelial cancer, Supportive care, Quality of life

## Abstract

**Background:**

Patient-reported outcomes (PROs) are getting widely implemented, but little is known of the impact of applying PROs in specific cancer diagnoses. We report the results of a randomized controlled trial (RCT) of the active use of PROs in patients with locally advanced or metastatic bladder cancer (BC) undergoing medical oncological treatment (MOT) with focus on determining the clinical effects of using PROs during chemo- or immunotherapy compared to standard of care.

**Methods:**

We recruited patients from four departments of oncology from 2019 to 2021. Inclusion criteria were locally advanced or metastatic BC, initiating chemo- or immunotherapy. Patients were randomized 1:1 between answering selected PRO-CTCAE questions electronically once weekly with a built-in alert-algorithm instructing patients of how to handle reported symptoms as a supplement to standard of care for handling of side effects (intervention arm (IA)) vs standard procedure for handling of side effects (control arm (CA)). No real-time alerts were sent to the clinic when PROs exceeded threshold values. Clinicians were prompted to view the completed PROs in the IA at each clinical visit. The co-primary clinical endpoints were hospital admissions and treatment completion rate. Secondary endpoints were overall survival (OS), quality of life (EORTC’s QLQ-C30 and QLQ-BLM30) and dose reductions.

**Results:**

228 patients with BC were included, 76% were male. 141 (62%) of the patients had metastatic disease. 51% of patients in the IA completed treatment vs. 56% of patients in the CA, OR 0.83 (95% CI 0.47–1.44, *p* = 0.51). 41% of patients in the IA experienced hospitalization vs. 32% in the CA, OR 1.48 (95% CI 0.83–2.65, *p* = 0.17). OS was comparable between the two arms (IA: median 22.3mo (95% CI 17.0-NR) vs. CA: median 23.1mo (95% CI 17.7-NR). Patient and clinician compliance was high throughout the study period (80% vs 94%).

**Conclusions:**

This RCT did not show an effect of PRO on completion of treatment, hospitalizations or OS for BC patients during MOT despite a high level of patient and clinician compliance. The lack of real-time response to alerts remains the greatest limitation to this study.

**Supplementary Information:**

The online version contains supplementary material available at 10.1186/s41687-023-00640-5.

## Introduction

Bladder cancer (BC) patients with advanced stages of disease have a poor prognosis [1, 2]. Only few oncological treatment possibilities exist and due to comorbidities, many patients are unfit for one or more of these treatment options [3–5]. From previous studies we have shown a high rate of hospital admissions and low rate of treatment completion for this patient group [6]. These poor clinical outcomes may to some extent be preventable, with the right intervention. One such intervention has over the past years been proposed to be the use of symptom questionnaires, patient-reported outcomes (PROs). The effect of real-time use of PROs to ensure timely handling of severe symptoms while in active oncological treatment has been tested in many settings with real-time responses and handling of symptoms either by the patients themselves or through added supportive care provided by study personnel [7–9]. For these purposes electronic PROs (ePROs) have been introduced to ensure completion of symptom questionnaires from home. Two studies by Denis and Basch testing the effect of the active use of PROs in different cancer populations on overall survival have shown an improved overall survival (OS) of 5–7 months compared to standard of care handling of symptoms and side-effects [10–12]. These data are of special interest to the BC population as a survival benefit of 5–7 months would markedly surpass the improvement in overall survival of 2–3 months seen for previously introduced 2nd line treatments [13]. However, PROs themselves have little or no clinical implications if not handled upon. Sole collection of PROs provides no direct benefit to the patient him- or herself [14]. Thus, the PROs must be used actively and generate a handling from either the patient herself or study personnel in order to secure the enhanced supported care. Also, the chosen PROs must be appropriate for the population and context to which they are applied [15–18]. We previously performed item selection specific to this group with the present study and endpoint in mind and found 15 appropriate symptoms explored by 30 items relevant for the present study [19]. Also, we tested the feasibility of ePRO use in the BC population and found a high questionnaire completion rate, even in this elderly and comorbid patient population [20].

In this study we report the effect of weekly ePROs as an intervention compared to standard of care for BC patients receiving chemo- or immunotherapy with the aim of reducing rate of hospital admissions during treatment and prevent early treatment cessation.

## Methods

### Patients

**Fig. 1:**
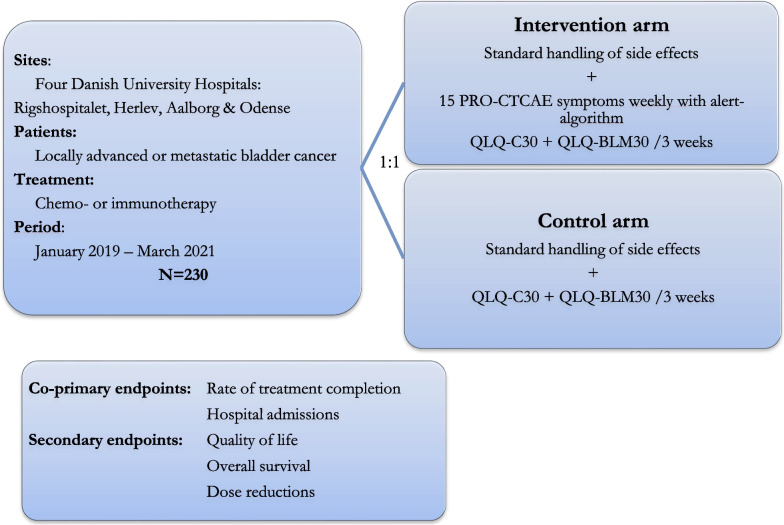
Study overview. Abbreviations: PRO-CTCAE: Patient Reported Outcomes version of the Common Terminology Criteria for Adverse Events. QLQ-C30: European Organisation for Research and Treatment of Cancers global quality of life core questionnaire. QLQ-BLM30: European Organisation for Research and Treatment of Cancers questionnaire for muscle-invasive bladder cancer. Randomization allocation was provided by the Ambuflex software

From 15th of January 2019 all patients with urothelial carcinoma of the bladder initiating chemo- or immunotherapy (cisplatin/gemcitabine, carboplatin/gemcitabine, pembrolizumab or vinflunin) as neoadjuvant treatment for muscle-invasive or locally advanced BC (from here on referred to as locally advanced BC) or palliative treatment for metastatic BC at the oncological department of four university hospitals (Copenhagen University Hospitals Rigshospitalet and Herlev Hospital, Aalborg University Hospital and Odense University Hospital) were asked to participate, please see Fig. 1. Further inclusion criteria were access to electronic communication with authorities through e-Boks™ as described in detail in a previous publication [20] and able to read Danish. Recruitment continued until 230 patients were enrolled.

Treatments (chemo- or immunotherapy) were for all patients given every three weeks in an out-patient setting. As part of enrollment into the study patients were asked to complete questionnaires (see below) for a maximum of six cycles of treatment [18 weeks]. Irrespective of allocated arm of randomization all patients could at any time contact their treatment department from home with troublesome symptoms.

The co-primary clinical endpoints were hospital admissions and treatment completion rate. Secondary endpoints were OS, quality of life (QoL) (EORTC’s QLQ-C30 and QLQ-BLM30) and dose reductions.

### The intervention arm

If allocated to the intervention arm (IA) patients received the first questionnaire the same day as initiating treatment by receiving an email in Denmark’s electronic platform for communication with authorities, e-Boks™. Ambuflex, a generic web-based PRO software developed in 2004 in Denmark, sends questionnaires to a patient through e-Boks™, with a link for the patient with the specific questionnaire [20, 21]. The frequency of questionnaires and interval between treatment cycles and accompanying clinical visits follows a fixed interval, as shown in Table 1.

**Table 1:** Frequency of questionnaires and clinical visits for intervention arm

Intervention arm	Baseline	Week 1	Week 2	Week 3	Ect
EORTC QLQ-C30	X			X	
EORTC QLQ-BLM30	X			X	
PRO-CTCAE	X	X	X	X	X
Clinical visits/ Treatment cycles	X			X	

If patients failed to complete the questionnaires a reminder was sent after two days.

During completion of the 15 PRO-CTCAE symptoms the patient was guided on-screen of how to handle the given symptom if the severity exceeded a predefined level of severity. The alert appeared as shown below in a blue box next to the question:

Symptoms were selected specifically for patients in chemo- or immunotherapy for urothelial cancer. The comprehensive process of selection is described in detail in a previous publication [19].

For all PRO-CTCAE items, alerts were prompted to the patient at a level of severity at which the study group (GT, HL, HP) agreed that the symptom in question should be handled, either by the patients themselves or by the treating department, Table 2. A predefined level of severity was defined to colour the ‘bars’ in the clinician view of the Ambuflex system reflecting the severity of the response, Table 2.

**Table 2:** PRO-CTCAE questions and threshold for on-screen alert for intervention arm

Symptom nb	Symptom	Items	Alert threshold PRO-CTCAE grade	Alert wording	Number of questions	Grade threshold yellow/red
1	Decreased appetite	Severity	2	2:*	1	2/ > 2
		Interference with daily activities	2	2:*	2	2/ > 2
2	Nausea	Frequency	1 + 2	1: If you have been given anti-emetics that you haven’t applied, please take them now. If in doubt, please contact the department 2: *	3	2/ > 2
		Severity	1 + 2	1: If you have been given anti-emetics that you haven’t applied, please take them now. If in doubt, please contact the department 2:*	4	2/ > 2
3	Vomiting	Frequency	1 + 2	1: If you have been given anti-emetics that you haven’t applied, please take them now. If in doubt, please contact the department 2: *	5	2/ > 2
		Severity	1 + 2	1: If you have been given anti-emetics that you haven’t applied, please take them now. If in doubt, please contact the department 2: *	6	2/ > 2
4	Constipation	Severity	1 + 2	1: If you have laxative medications at home but are in doubt of how to apply them, please contact the department 2:*	7	2/ > 2
5	Diarrhea	Frequency	1	1:*	8	2/ > 2
6	Shortness of breath	Severity	2**	2:*	9	2/ > 2
		Interference with daily activities	2**	2:*	10	2/ > 2
7	Swelling	Frequency	2	2:*	11	2/ > 2
		Severity	2	2:*	12	2/ > 2
		Interference with daily activities	2	2:*	13	2/ > 2
8	Heart palpitations	Frequency	2	2:*	14	2/ > 2
		Severity	2	2:*	15	2/ > 2
9	Itching	Severity	2	2:*	16	> 1
10	Pain	Frequency	1 + 2	1: If you have pain medication at home and have doubts of how to apply them, please call the department 2: *	17	2/ > 2
		Severity	1 + 2	1: If you have pain medication at home and have doubts of how to apply them, please call the department 2: *	18	2/ > 2
		Interference with daily activities	1 + 2	1: If you have pain medication at home and have doubts of how to apply them, please call the department 2: *	19	2/ > 2
11	Insomnia	Severity	2	2:*	20	2/ > 2
		Interference with daily activities	2	2:*	21	2/ > 2
12	Fatigue	Severity	3	3:*	22	2/ > 2
		Interference with daily activities	3	3:*	23	2/ > 2
13	Anxiety	Frequency	2	2:*	24	2/ > 2
		Severity	2	2:*	25	2/ > 2
		Interference with daily activities	2	2:*	26	2/ > 2
14	Frequent urination	Frequency	3	3:*	27	3/ > 3
		Interference with daily activities	3	3:*	28	3/ > 3
15	Chills	Frequency	1	1:*	29	2/ > 2
		Severity	1	1:*	30	2/ > 2

^*^: Please contact the department at which you’re treated. Remember to complete the rest of the questionnaire, even though you contact the department. **: or at deterioration from baseline

**Fig. 2:**
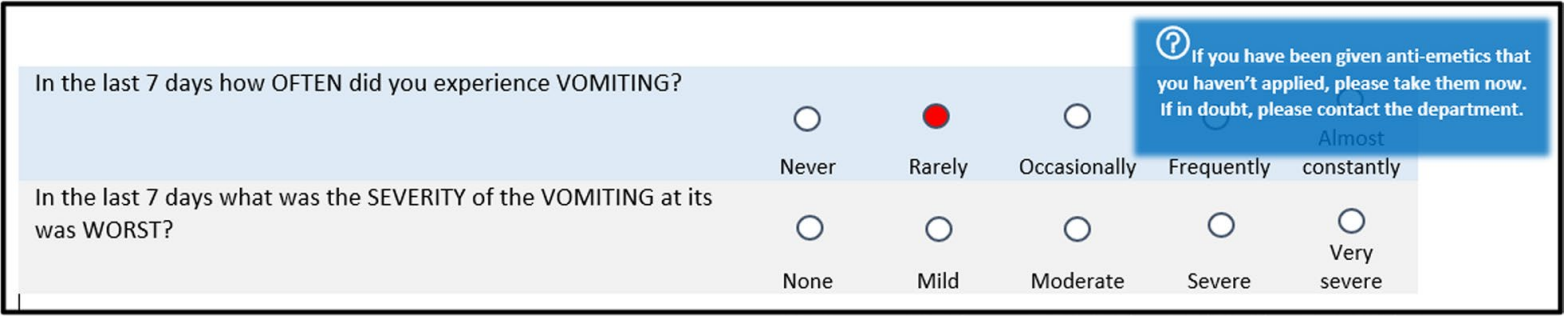
Patient view of questions, i.e. exploring the symptom ‘vomiting’ and example of alert (blue box). The above example is a translation of the patient view which was in Danish

**Fig. 3:**
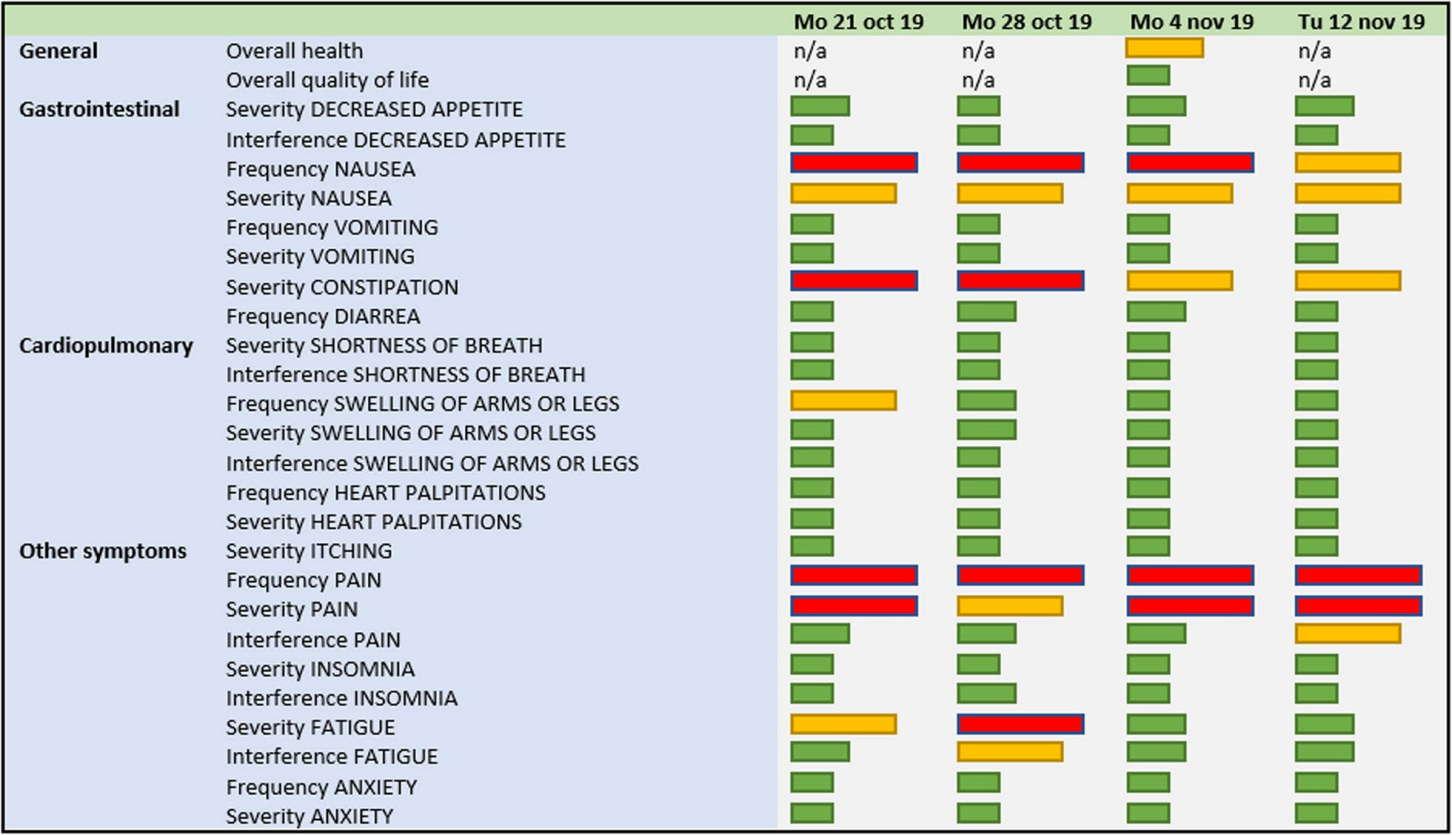
Clinician view of patient responses with coloured bars according to severity of a given symptom

For patients in the IA the treating clinician were at all following clinical visits reminded of responses in the Ambuflex system (Fig. 2) and could use this information in the conversation and treatment of the patient. As such, while the patients in the intervention arm completed questionnaires weekly, the clinicians were only prompted to view the development of the symptoms in Fig. 2 at clinical visits planned for every third week according to treatment cycles. No real-time alerts were sent to the clinic when PROs exceeded threshold values. Patients were thus expected to act upon the on-screen alerts, as illustrated in Fig. 3 and Table 2.

In response to the PROs, the clinician was not given a predefined course of action depending on the symptom and severity thereof. It was at the discretion of the clinician to use these data as he or she saw fit.

In order to evaluate the use of the above-described intervention in the daily clinic, we aimed to estimate the percentage of clinical visits in which the questionnaire was viewed by a clinician. We reviewed all clinician logs into Ambuflex coinciding with a completed questionnaire and divided the number by number of completed cycles of treatment for the IA.

### The control arm

Patients assigned to the control arm (CA) followed standard procedure for handling of side effects and symptoms as informed by the treating department. The patients completed QoL questionnaires once every three weeks, as shown in Table 3. No on-screen alerts in response to these questionnaires were given and the clinicians were not, as in the IA, made aware of responses in Ambuflex when the patient came for clinical visits at the hospital. The completed questionnaires, although present in Ambuflex, were not presented to the clinician with coloured bars as in the IA.

**Table 3:** Frequency of questionnaires and clinical visits for control arm

Control arm	Baseline	Week 3	Week 6	Week 9	Ect
EORTC QLQ-C30	X	X	X	X	X
EORTC QLQ-BLM30	X	X	X	X	X
Clinical visits/treatment cycles	X	X	X	X	X

### Statistical analysis

On the basis of rates of treatment completion from a previous study and literature review, the current study was planned to include 230 patients. Prior data indicated that the rate of treatment cessation among controls was 50% [22–24] and data from our previous study indicated a treatment cessation rate of 54% [6]. If the true rate of treatment cessation for experimental subjects was 30% we would need 103 experimental subjects and 103 control subjects to be able to reject the null hypothesis that the failure rates for experimental and control subjects were equal with probability (power) 0.8. Allowing for expected attrition, 230 patients were planned for inclusion, 115 patients in each group. The type I error probability associated with this test of this null hypothesis was 0.05.

The proportion of patients experiencing early treatment cessation, hospitalization or dose reduction was compared between arms using Fisher’s exact test. OS was defined as time from inclusion in the study to death from any cause. The survival curves were calculated using the Kaplan–Meier estimator and compared between arms using the log-rank test. Median OS (mOS) was computed based on the estimated survival curves. The Cox proportional hazards model was used to estimate hazard ratios and corresponding 95% CIs. Variables with possible effect on survival were included in the analyses. Differences between arms in QoL was assessed using linear mixed effects models with time and group as fixed effects allowing for interaction, and patient as a random effect. Minimal important differences (MID) for interpreting EORTC QLQ-C30 were applied according to Musoro et al. and a MID of 10 point was applied in the interpretation of the results [25] The statistical analysis was carried out using R version 4.2.1 [26].

### Ethical considerations

The study was approved by the Danish Data Protection Agency (suite nb: RH-2017–348), registered at www.clinicaltrials.gov with NCT03584659 and all patients completed written informed consent before entry into the study and randomisation. According to Danish law concerning sole questionnaire intervention studies at the time of study conduction, the study was exempt from approval from the National Committee on Health Research Ethics. The study was checked against the CONSORT Statement list of recommendations for randomized controlled trials with patient reported outcomes and the PRO extension, please see (Additional file 1: Table 1)  [27].

## Results

**Fig. 4:**
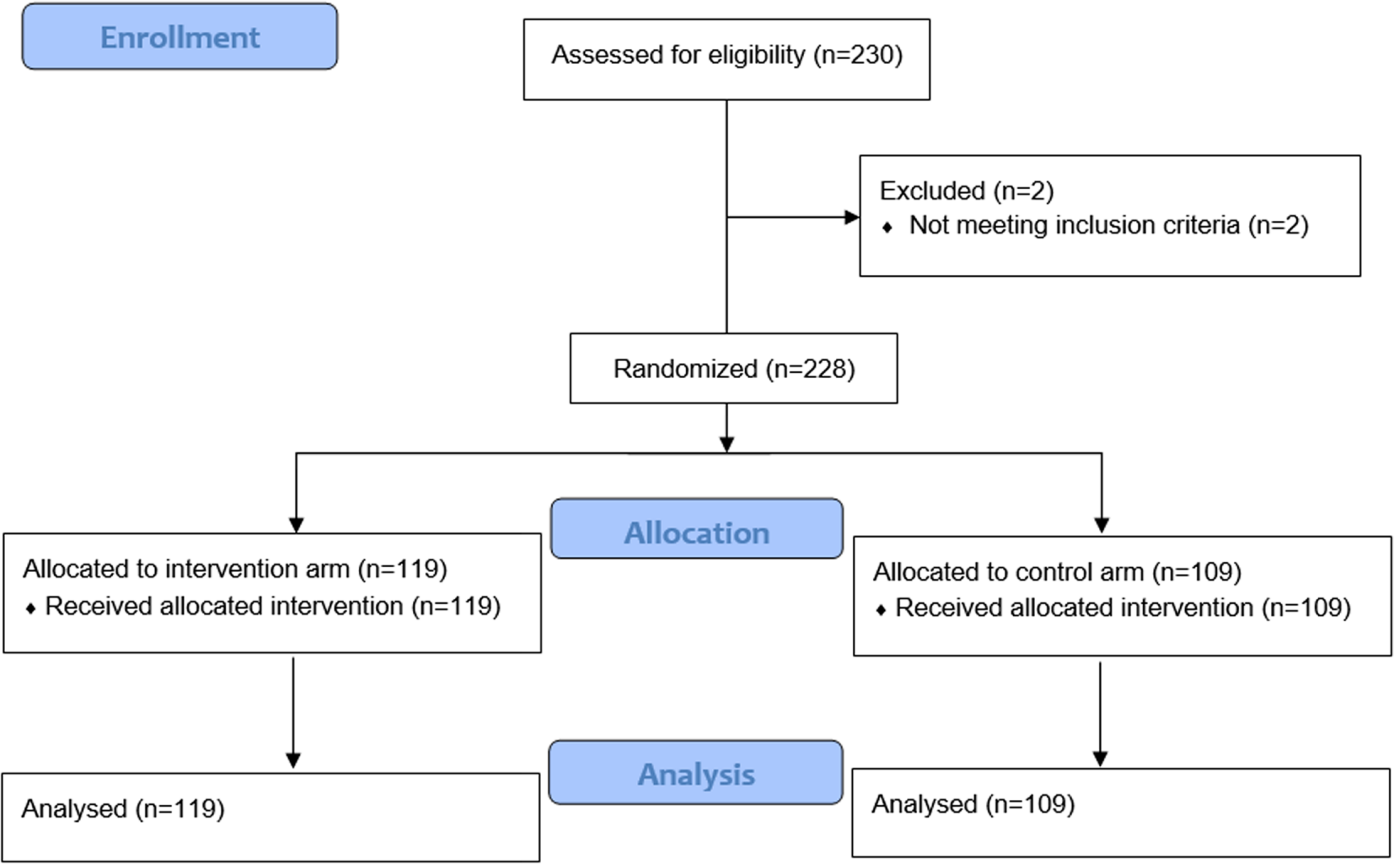
CONSORT diagram of patients enrolled

From 22nd January 2019 to 19th March 2021, we enrolled a total of 230 patients. Two patients were later excluded due to final pathology report requiring a different treatment than that of our inclusion criteria and another did not initiate treatment. The analysis therefore included 228 patients, Fig. 4.

The clinical data of the 228 patients are shown Table 4. The clinical characteristics of the patients were in concordance with other published studies of the bladder cancer cohorts and evenly dispersed between the two groups, with no significant differences [5, 28, 29]. Similar, no differences between groups are seen regarding stage of disease or systemic treatment modality. Median follow-up time from randomization to registration of death vs alive was 14.1 months.

**Table 4:** Clinical data

Clinical data	All patients N = 228	Intervention arm N = 119	Control arm N = 109
*Gender*			
Male, n (%)	173 (76)	90 (76)	83 (76)
Female, n (%)	55 (24)	29 (24)	26 (24)
Median age (years, range)	69 (40–87)	69 (44–87)	68 (40–86)
*Disease stage*			
Locally advanced, n (%)	87 (38)	47 (40)	40 (37)
Metastatic, n (%)	141 (62)	72 (60)	69 (63)
*Treatment*			
Cisplatin + gemcitabine, n (%)	135 (59)	70 (59)	65 (60)
Carboplatin + gemcitabine, n (%)	36 (16)	19 (16)	17 (16)
Vinflunine, n (%)	1 (0)	1 (1)	0 (0)
Pembrolizumab, n (%)	56 (25)	29 (24)	27 (25)

**Fig. 5:**
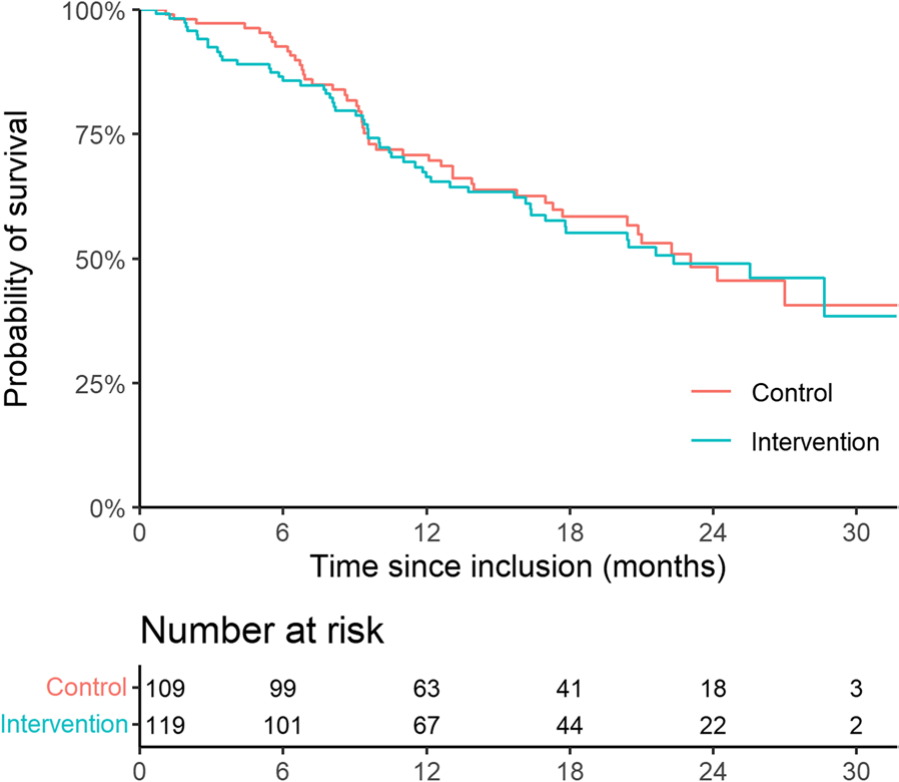
Kaplan–Meier curve of overall survival in both study groups

We observed no difference in rate of hospital admissions (OR 1.48, 95% CI 0.83–2.65, *p* = 0.17) or completion of treatment (OR 0.83, 95% CI 0.47–1.44, *p* = 0.51) between the two groups, Table 5. Likewise, no difference in OS was found (mOS intervention arm: 22.3 months (95% CI 17.0-NA) vs. mOS control arm: 22.1 months (95% CI 17.7-NA)), *p* = 0.8, Table 5 and Fig. 5. Upper level of the confidence interval could not be computed due to too few deaths in the study period. When looking at OS with the clinical characteristics as covariates in a Cox proportional hazards model, only disease stage was found significant for survival (HR 4.91 (95% CI 2.62–19, *p* < 0.0001), Table 6. No difference in rate of dose reductions was observed between the two study arms; 19% vs 17%, OR 1.21 (95% CI 0.58–2.55, *p* = 0.61).

**Table 5:** Endpoints. NA: It was not possible to find upper confidence levels for median overall survival due to too few deaths during the study period

Endpoints	All patients N = 228	Intervention arm N = 119	Control arm N = 109	P-value
Hospital admissions, n (%)	84 (37)	49 (41)	35 (32)	0.17
Completion of treatment, n (%)	122 (56)	61 (51)	61 (56)	0.51
Overall survival, (months median, 95% CI)	22.3 (20.4-NA)	22.3 (17.0-NA)	23.1 (17.7-NA)	0.8
Dose reductions, n (%)	41 (18)	23 (19)	18 (17)	0.61

**Table 6:** Cox proportional hazards model for survival

Cox proportional hazards model for survival	HR	CI	*p*-value
Study arm			
Intervention	1		
Control	0.94	0.63–1.41	0.77
Gender			
Female	1		
Male	0.75	0.48–1.17	0.2
Age (per 1 yr increase)	0.99	0.97–1.02	0.83
Treatment			
Carboplatin/gemcitabine	1		
Cisplatin/gemcitabine	0.75	0.41–1.38	0.36
Pembrolizumab	0.75	0.43–1.31	0.31
Vinflunine	0.66	0.09–4.95	0.68
Disease stage			
Locally advanced	1		
Metastatic	4.91		< 0.0001

**Fig. 6:**
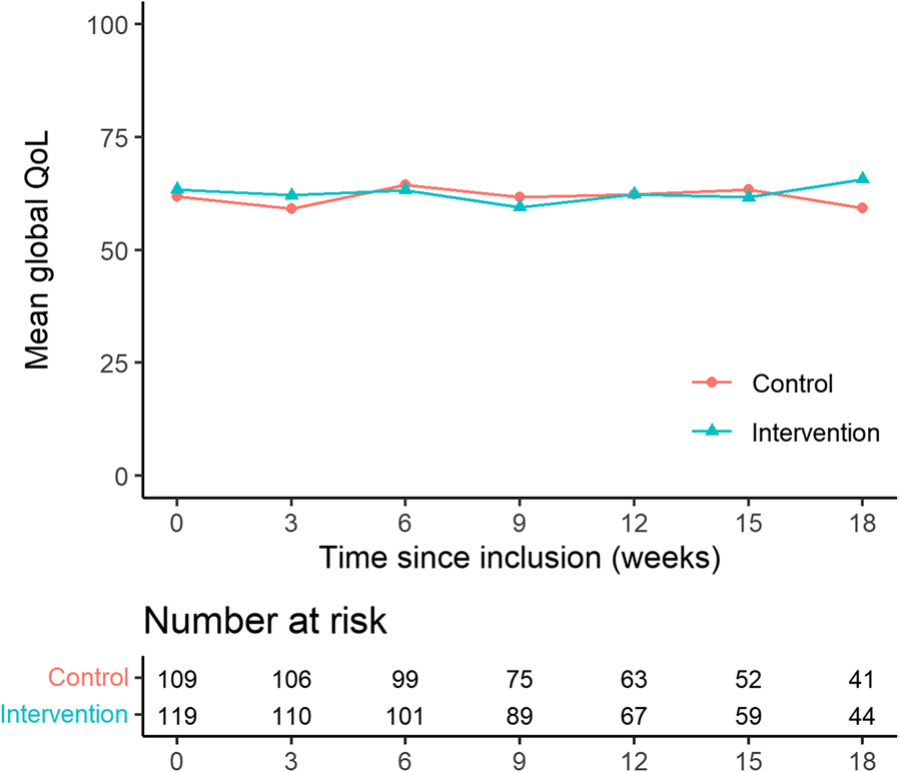
Mean global quality of life over the course of the study

**Fig. 7:**
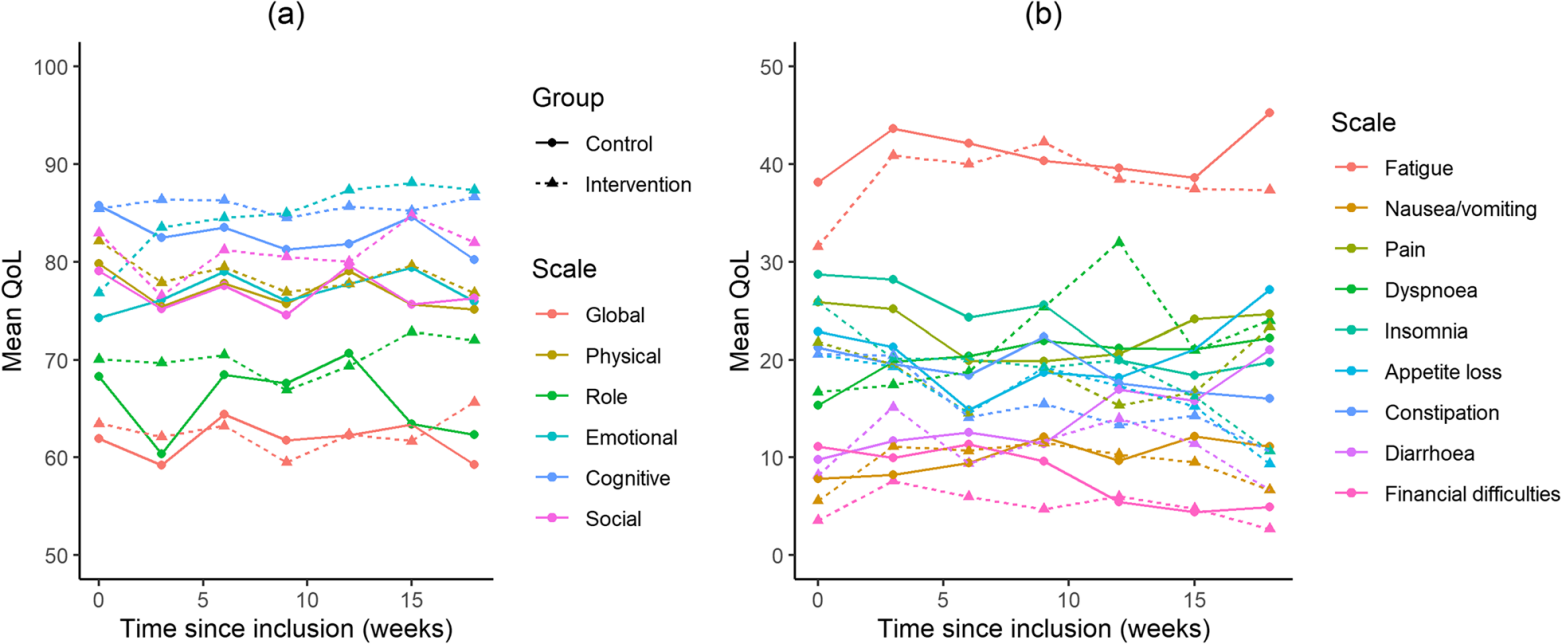
Mean quality of life over the course of the study. **a** Global- and functional scales, **b** symptom scales

Global QoL for all patients was stable over time (Mean Global QoL 61–63 (SD: 22–26)). At the end of the study period global QoL increased for the intervention arm while the control arm experienced a decrease in QoL, although this difference was not statistically significant, Table 7 and Fig. 6. When looking at the subscales of QoL the data showed a highly significant and clinically meaningful difference in emotional functioning between the intervention and control arm favouring the intervention arm (Point estimate at week 18 = 13.6, *p* = 0.0001) [25]. No differences between arms were found for the remaining QoL subscales, Fig. 7.

**Table 7:** Quality of life and questionnaire completion over time

Global quality of life	All patients N = 228	Intervention arm N = 119	Control arm N = 109
*Quality of life, mean (SD)*			
Baseline	63 (22)	63 (22)	62 (23)
After 3 cycles (9 weeks)	61 (22)	60 (23)	62 (20)
After 6 cycles (18 weeks)	62 (26)	66 (24)	59 (27)
*Questionnaire completion*			
Baseline	86%	79%	94%
After 3 cycles (9 weeks)	75%	71%	78%
After 6 cycles (18 weeks)	65%	64%	66%

**Fig. 8:**
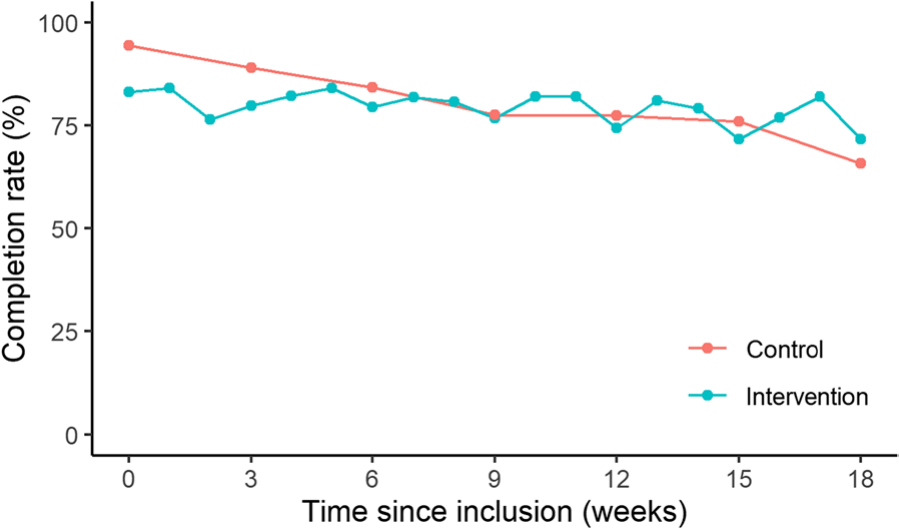
Questionnaire completion rate over time for both arms

Completion rate of electronic questionnaire for all patients was 69–88% through the course of 0–18 weeks with the highest completion rate (88.6%) at week 0. The completion differed between the two groups, as illustrated by Fig. 8. Completion rate declined steadily over time for patients in the control arm whereas completion for patients in the intervention arm remained at a stable level throughout treatment but repeatedly declined every third week coinciding with the longer questionnaire (EORTC QLQ-C30, QLQ-BLM30 and PRO-CTCAE) whereas completion remained above 80% for the short questionnaires (PRO-CTCAE).

The level of clinician compliance was determined by number of logs into the Ambuflex system coinciding with a completed questionnaire and compared with number of planned visits (as expected according to number of completed cycles of treatment). We found that the intervention group had a total of 495 completed cycles of treatment. The number of clinician logs into Ambuflex for the intervention group was 466, equaling a clinician compliance of 94%. However, against the intention of the study, we also found a clinician log into Ambuflex for the control group of 240. The control group had a total of 467 completed cycles equaling an unintended clinician log into Ambuflex (which did not show any listing of EORTC or PRO-CTCAE data) of 51%.

## Discussion

In this multicenter randomized trial testing the active use of PROs during systemic oncological treatment for BC patients, we found a high rate of completed questionnaires and a high rate of clinician viewing of the patient-reported symptoms. For the primary endpoints, no statistically significant differences were found, although a higher rate of hospital admissions and lower rate of treatment completion was found in the IA. During the study period, global QoL increased for the IA and decreased for the CA, however these finding were not statistically significant. Emotional functioning was significantly higher for the IA. No differences were found in OS between the two study arms.

Unlike previously reported PRO trials testing the impact of systematic symptom reporting and enhanced handling of side effects, our study was unable to demonstrate differences in the chosen endpoints. Many explanations for this may exist. A major difference between ours and previous studies testing the use of PRO for cancer patients in treatment is that previously reported studies have implemented study staff to, on a daily basis, react upon alarming symptoms reported from home and then contacting the patient to initiate the appropriate supported care [11, 22, 30, 31]. Specifically, in a study by Maguire et al., alarming symptoms required study personnel attention within 30 min to 8 h. Hospital clinicians received these alerts on dedicated handsets and a predefined algorithm of how to handle a given symptom guided the clinical staff of how to handle the symptom in collaboration with the patient at home [30]. In the present study we relied on the patient following the on-screen instructions when exceeding a certain limit, as defined in Table 2. This patient-led strategy has been described effective for enhancing physical well-being and self-efficacy in the eRAPID study conducted in a health care system similar to the Danish health care system [32]. Low health literacy and/or unclear instructions for engaging the patients in the self-efficacy needed to act upon guidance shown on-screen may cause low impact of the tested intervention [33, 34], although health literacy in Denmark generally is perceived high compared to other European countries [35]. Insufficient training of the patients prior to PRO completion from home may also have affected our results. Recently, a study by Mooney et al. showed the importance of multicomponent interventions highlighting the importance of all components of PRO handling and interventions, thus showing lower effect on symptom relief with fewer interventional components [36]. Therefore, we may have missed opportunities to show significant effects in the chosen endpoints by solely relying on the patient to act upon alerts presented while PRO reporting from home.

In this study we found a mean questionnaire completion rate of 80% through the study period which is at level with previously conducted PRO studies [37–39]. This high compliance would along with the improved emotional functioning seen for the intervention group be assumed sufficient to enable engagement with the treating clinician at every clinical visit and support the self-efficacy needed to act upon on-screen guidance when prompted to do so as a result of a given symptom exceeding the predefined threshold [7, 30, 40]. The negative findings could therefore pinpoint the necessity of daily study personnel to support the intervention.

Clinician reluctance to the use of PROs and the importance of clinician endorsement to achieve effects of PRO have been described in several previous publications [41–43]. We found very high clinician compliance (94%) for the IA which explains the continuously high completion rate for the patients as seen for the IA in Fig. 8. However, we also saw clinician logs into the Ambuflex system for the CA of 51%, although no PRO data could be found as seen in Fig. 2 for the IA. Our inability to demonstrate a difference between the two study arms may therefore be a so-called spillover effect of the intervention to the control group [44]. Thus, the enhanced awareness on symptoms during treatment for both groups may have clouded for the actual impact in the intervention group by bringing enhanced symptom handling to both groups, unintentionally.

Although not statistically significant, we found a higher rate of hospitalizations and lower rate of treatment completion in the intervention group when compared with the control group. Though unintentional to the initial hypothesis and aim of the study, the enhanced focus on symptom handling and toxicity may have led to an increase in hospitalizations and earlier treatment cessations. In a previous study in the breast cancer population increased awareness to toxicity was previously described to lead to early treatment cessation [45]. However, in the study by Basch et al. from 2017, fewer hospitalizations were seen for the patients in the intervention arm, although this population of patients only comprised patients in treatment with chemotherapy [22]. The introduction of immunotherapy has led to the introduction of worldwide toxicity algorithms guiding clinicians to handle toxicities to treatments alike despite different hospital settings [46]. These pre-defined algorithms for toxicity handling may over time and during our study period have enhanced attention to side effects thus minimizing the potential benefit aimed for according to our power calculations performed with data from the pre-immunotherapy era. The PRO-TECT study by Basch et al. from 2022 included patients receiving immunotherapy and demonstrated a significant effect on symptom control and HRQOL with similar effect sizes as our study but did not report the impact on hospitalizations or treatment adherence. The PRO-TECT study was in addition to patient self-management advice planned with alerts to clinical staff on a daily basis and may explain the differences in our findings [31, 36].

Despite a small increase in global quality of life was observed for the intervention group compared to the control group we did not find a statistically significant difference between the two groups. However, a randomized study with an ePRO intervention in a population of metastatic melanoma patients showed that quality of life between the two arms did not separate until months after the applied ePRO intervention [47]. In the present study, we did not measure quality of life after patients had ended treatment, mainly of ethical reasons as patients who ceased treatment often did so due to troublesome symptoms, hospitalization or progressive disease and death. Had the patients who completed 6 cycles of treatment continued QoL reporting we may have found a clinically meaningful and statistically significant difference post-intervention.

Strengths of the study include the randomized trial design conducted at four university hospitals across Denmark. Also, the extensive pilot studies leading to this design, in terms of choice of endpoints and item selection process involving patients, nurses, physicians and review of literature is a major strength of this study. The conduction of the pilot studies spread over 1½ year allowing for familiarity with PROs as a part of daily clinic was in this study reflected in the high level of clinician compliance.

The lack of multicomponent handling of PROs exceeding threshold values to clinicians to allow for real-time handling of symptoms remains the greatest limitation in terms of not reaching the specified endpoints. Also, a limitation of the study may have been that no standard procedures were given to clinicians of how to handle a given side effect as reported by the patient in the ePROs. The study was planned as such to allow for individual physician-led treatment of a given side effect. However, there may at one or more sites have been a high clinical standard of handling of side effects before study initiation, thereby diminishing the impact of the intervention. The lack of this guidance specific to this study may across four study sites have led to vast differences in the handling of side effects for both arms in the study. In a study by Maguire et al. from 2021 they achieved improvements in anxiety, health related quality of life, self-efficacy and supportive care needs in the intervention group despite the multinational setup and may be explained by their extensive symptom management flow charts [30]. We did not track clinician response to troublesome PROs as a result of the clinical visit and viewing of PROs and thus we do not know whether this component of our intervention had the intended effect on symptom handling.

## Conclusions

Conclusively, we did not find ePROs effective for the bladder cancer patients in relation to the chosen endpoints. We observed a high level of clinician engagement in using the ePROs and a positive impact on patients’ emotional functioning. Our study demonstrates the caveats in applying PROs across patient groups as a result of the increased awareness to PROs over the past years. For the bladder cancer patients with limited resources this approach may be unnecessarily time consuming in what sparse time left. The study group continues to evaluate the collected PRO data in order to find subsets of symptoms indicative for one or more of the chosen endpoints. Thus, further analyses will be made with the overall aim of improving the clinical courses of patients with bladder cancer.

### Supplementary Information

**Additional file 1:** Information for reporting randomized controlled trials with patient reported outcomes.

## Data Availability

The data that support the findings of this study are available from Gry Assam Taarnhøj but restrictions apply to the availability of these data, which were used under license for the current study, and so are not publicly available. Data are however available from the authors upon reasonable request and with permission of Gry Assam Taarnhøj.

## Abbreviations

BC: Bladder cancer
CA: Control arm
CI: Confidence interval
EORTC: European Organisation for Research and Treatment of Cancer
ePRO: Electronic patient-reported outcomes
HR: Hazard ratio
IA: Intervention arm
MOT: Medical oncological treatment
mOS: Median overall survival
OS: Overall survival
PRO: Patient-reported outcome
PRO-CTCAE: Patient-reported outcomes version of the common terminology criteria for adverse events
QLQ-BLM30: EORTCs quality of life module for invasive bladder cancer
QLQ-C30: EORTCs general core quality of life module
QoL: Quality of life
RCT: Randomized controlled trial
SD: Standard deviation
