# Supplementary material for: The iBLAD study: patient-reported outcomes in bladder cancer during oncological treatment: a multicenter national randomized controlled trial
Source: J Patient Rep Outcomes. 2023 Oct 9;7:99. doi: 10.1186/s41687-023-00640-5 (PMC10562329; doi:10.1186/s41687-023-00640-5)
Supplement: Supplementary file 1 — Additional file 1: Information for reporting randomized controlled trials with patient reported outcomes. [file 41687_2023_640_MOESM1_ESM.docx]

| Table 1. Information for Reporting Randomized Controlled Trials With Patient reported Outcomes | | | | |
| --- | --- | --- | --- | --- |
| **Section/Topic** | **Item** | **CONSORT 2010 Statement checklist item** | **PRO-specific Extensions Are Prefaced by the letter P** | **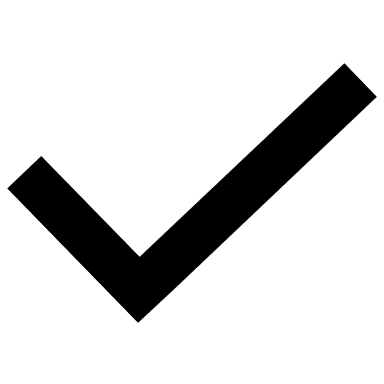** |
|  | 1a | Title and abstract: Identification as a randomized trial in the title |  | **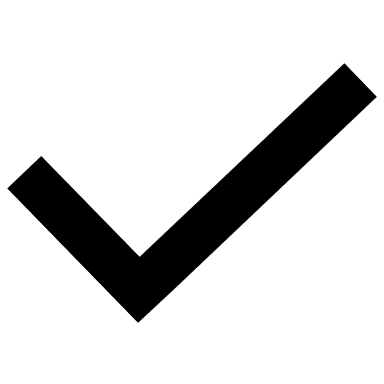** |
|  | 1b | Structured summary of trial design, methods, results and conclusions | P1b: The PRO should be identified in the abstract as a primary or secondary outcome | **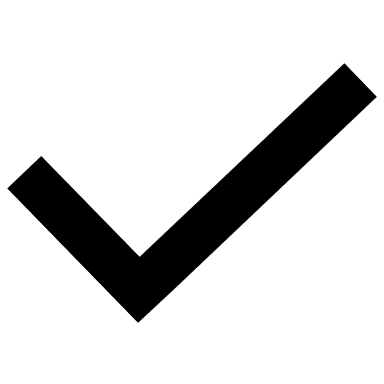** |
| Background and objectives | 2a | Introduction: Scientific background and explanation of rationale | Including background and rationale for PRO assessment | **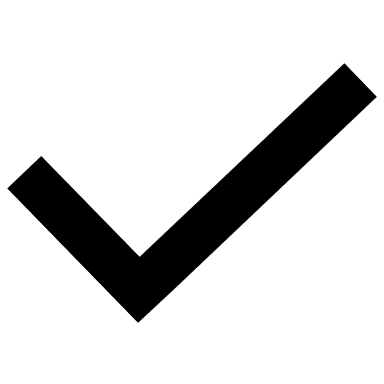** |
|  | 2b | Specific objectives or hypotheses | P2b: The PRO hypothesis should be stated and relevant domains identified, if applicable | **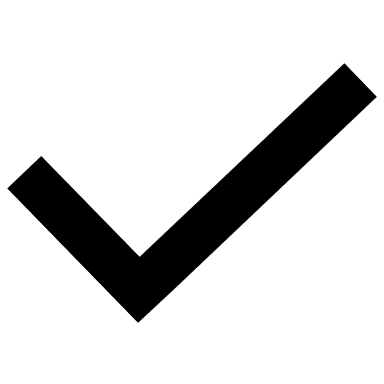** |
| Trial design | 3a | Methods: Description of trial design |  | **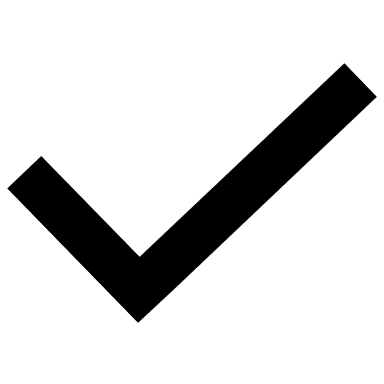** |
|  | 3b | Important changes to methods after trial commencement |  | N/A |
| Participants | 4a | Eligibility criteria for paticipants | Not PRO-specific, unless the PROs were used in eligibility or stratification criteria | **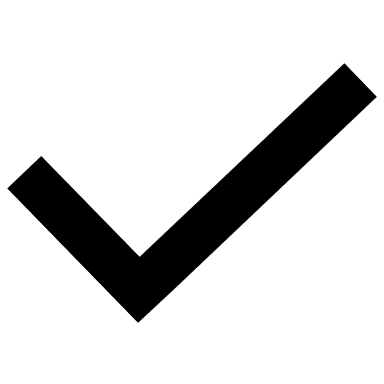** |
|  | 4b | Settings and locations where the data were collected |  | **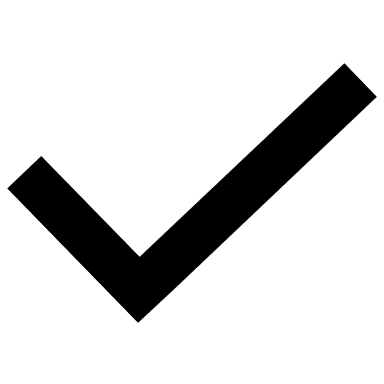** |
| Interventions | 5 | The interventions for each group with sufficient details to allow replication, including how and when they were actually administered |  | **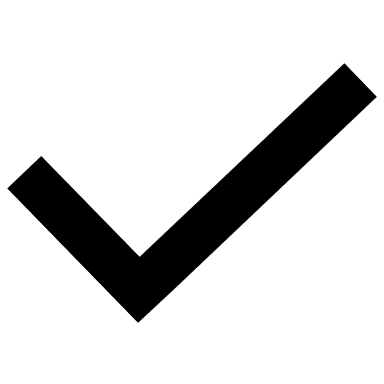** |
| Outcomes | 6a | Completely definded prespecified primary and secondary outcome measures, including how and when they were assessed | P6a: Evidence of PRO instrument validity and reliability should be provided or cited if available including the person completing the PRO and methods of data collection | **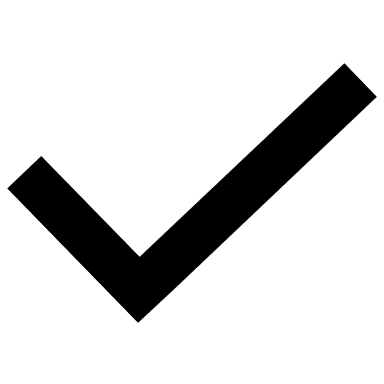** |
|  | 6b | Any changes to trial outcomes after the trial commenced with reasons |  | N/A |
| Sample size | 7a | How sample size was determined | Not required for PRO unless it is a primary study outcome | **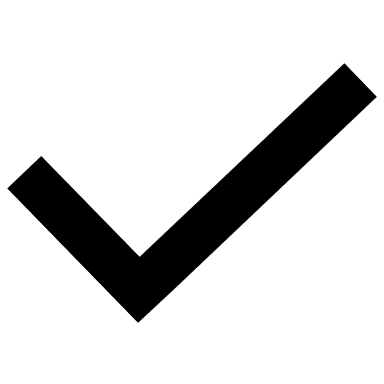** |
|  | 7b | When applicable, explanations of any interim analyses and stopping guidelines |  | N/A |
| Sequence generation | 8a | Randomization: Method used to generate the random allocation sequence |  | **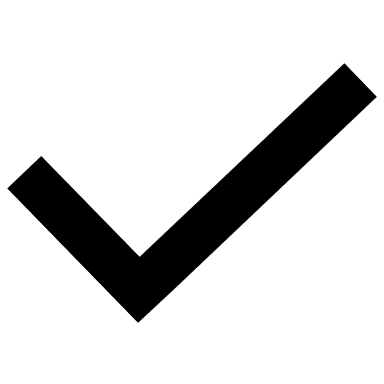** |
|  | 8b | Type of randomization: details of any restriction |  | N/A |
| Allocation concealment mechanism | 9 | Mechanism used to implement the random allocation sequence, describing any steps taken to conceal the sequence until interventions were assigned |  | N/A |
| Implementation | 10 | Who generated the random allocation sequence, who enrolled participants, and who assigned participants to interventions |  | **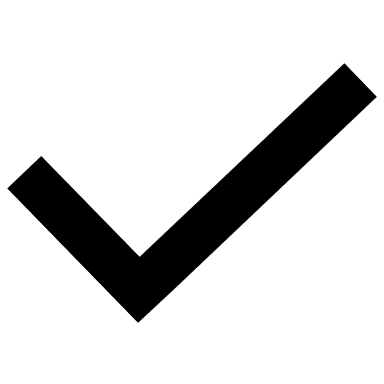** |
| Blinding | 11a | If done, who was blinded after assignment to interventions and how |  | N/A |
|  | 11b | If relevant, description of the similarity of interventions |  | **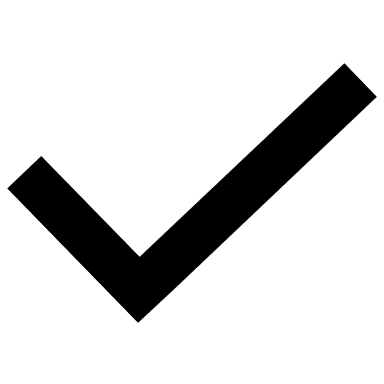** |
| Statistical methods | 12a | Statistical methods used to compare groups for primary and secondary outcomes | P12a: Statistical approaches for dealing with missing data are explicitly stated. | **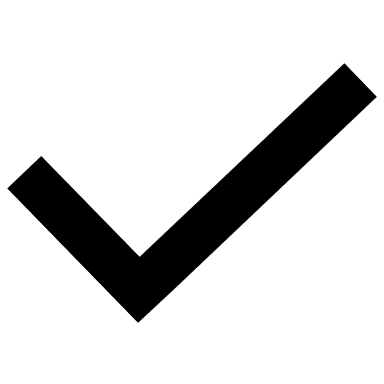** |
|  | 12b | Methods for additional analyses, such as subgroup analyses and adjusted analyses |  | **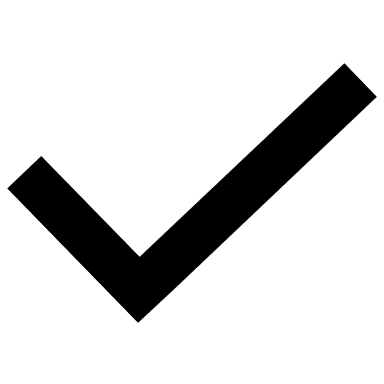** |
| Participant flow (a diagram is strongly recommended) | 13a | Results: For each group, the numbers of participants who were randomly assigned, received intended treatment, and were analysed for the primary outcome | The number of PRO outcome data at baseline and at subsequent time points should be made transparent. | **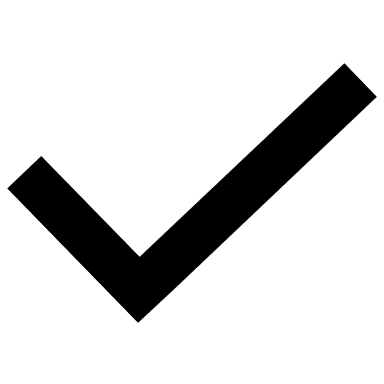** |
|  | 13b | For each group, losses and exclusions after randomization, together with reasons |  | **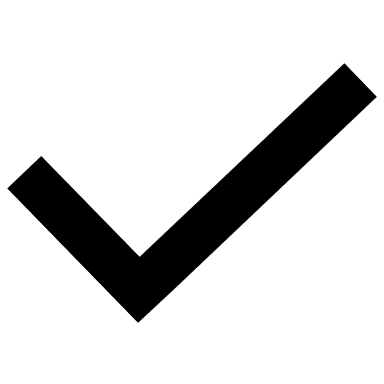** |
| Recruitment | 14a | Dates defining the periods of recruitment and follow up |  | **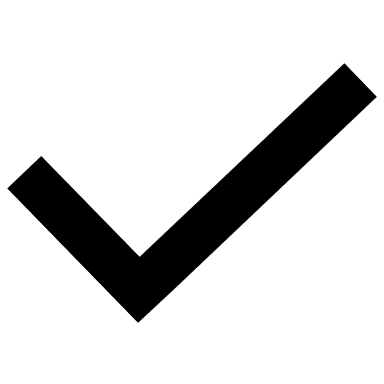** |
|  | 14b | Why the trial ended or was stopped |  | **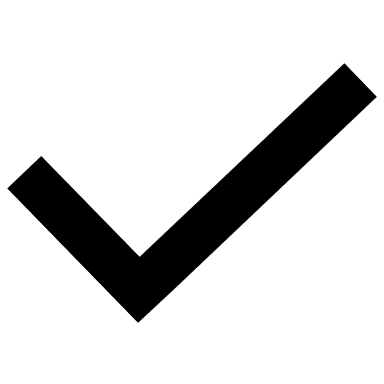** |
| Baseline data | 15 | A table showing baseline demographic and clinical characteristics for each group | Including baseline PRO data when collected | **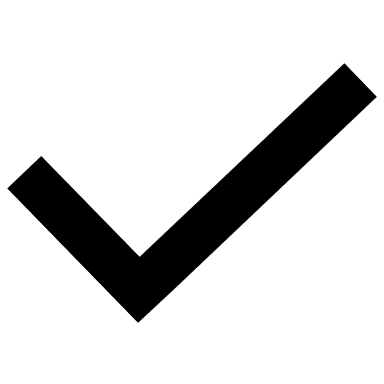** |
| Numbers analysed | 16 | For each group, number of participants included in each analysis and whether the analysis was by original assigned groups | Required for PRO results | **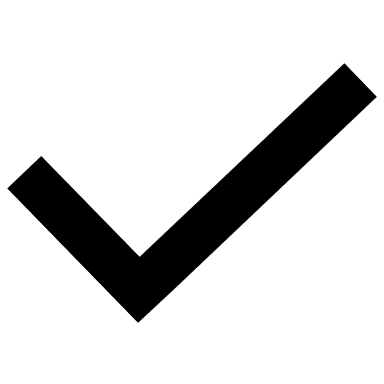** |
| Outcomes and estimation | 17a | For each primary and secondary outcome, results for each group, the estimated effect size, and its precision | For multidimensional PRO results from each domains and time point | **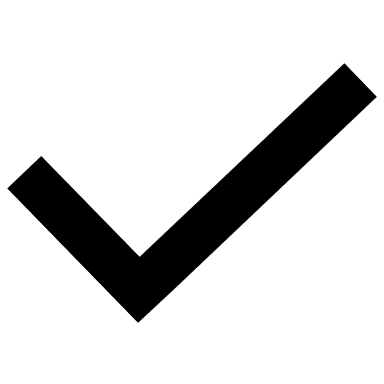** |
|  | 17b | For binary outcomes, presentation of both absolute and relative effect sized is recommended |  | **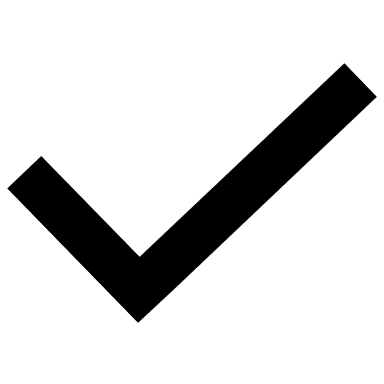** |
| Ancillary analyses | 18 | Results of any other analyses performed, including subgroup analyses and adjusted analyses, distinguishing prespecified from exploratory | Including PRO analyses, where relevant | **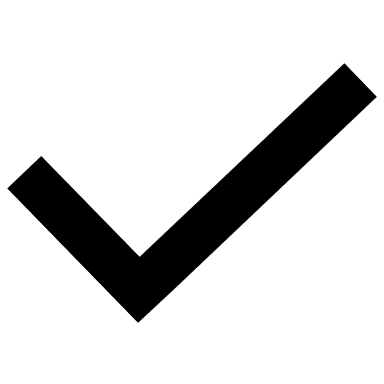** |
| Harms | 19 | All important harms or unintended effects in each group |  | **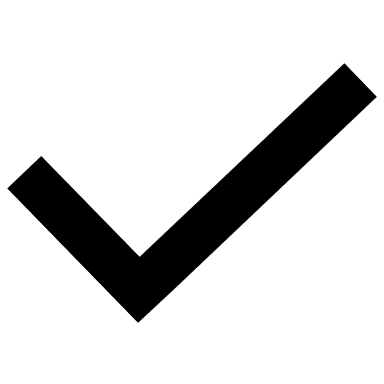** |
| Limitations | 20 | Discussion: Trial limitations, addressing sourced of potential bias, imprecision, and, if relevant, multiplicity of analyses | P20/21: PRO-specific limitations and implications for generalizability and clinical practice | **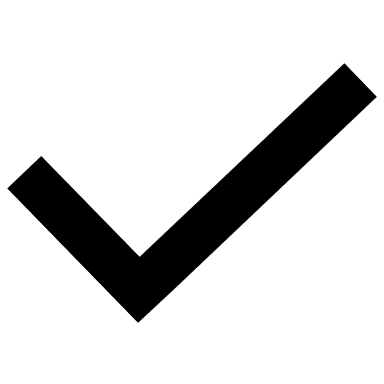** |
| Generalizability | 21 | Generalizability of the trial findings |  | **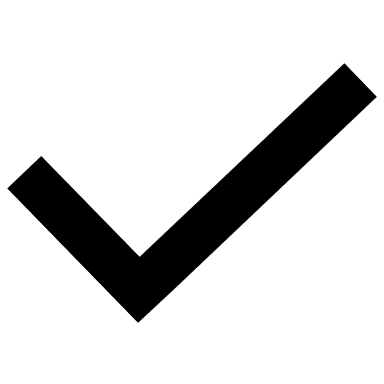** |
| Interpretation | 22 | Interpretation consistent with results, balancing benefits and harms, and considering other relevant evidence | PRO data should be interpreted in relation to clinical outcomes including survival data, where relavant | **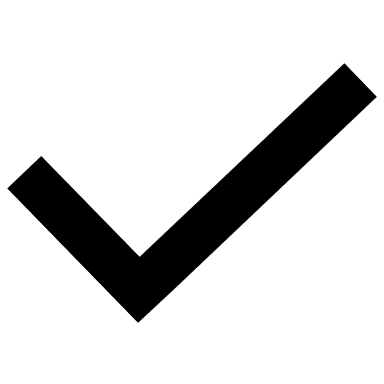** |
| Registration | 23 | Registration number and name of trial registry |  | **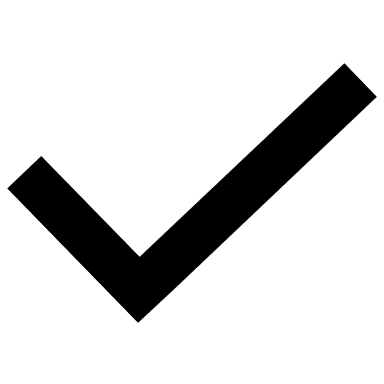** |
| Protocol | 24 | Where the full trial protocol can be accessed, if available |  | N/A |
| Funding | 25 | Sources of funding and other support |  | **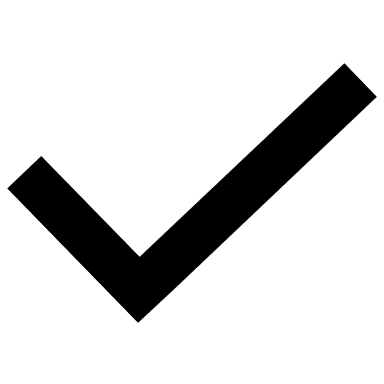** |

Reproduced from Reporting of Patient-Reported outcomes in Randomized Trials: The CONSORT PRO Extension. JAMA, February 27, 2013_Vol309(8)
